# Exploring the Cytotoxic and Anticancer Potential of Naringin on Oral Cancer Cell Line

**DOI:** 10.7759/cureus.64739

**Published:** 2024-07-17

**Authors:** Beniel Vincer, Jospin Sindya, Jeevitha Rajanathadurai, Elumalai Perumal

**Affiliations:** 1 Cancer Genomics Laboratory, Saveetha Medical College and Hospitals, Saveetha Institute of Medical and Technical Sciences, Saveetha University, Chennai, IND

**Keywords:** anticancer, anticancer drug discovery, oral cancer cell line (kb-1), oral cancer therapy, oral cancer and oncology, oral cancers, mtt assay, phytochemical compounds, naringin

## Abstract

Introduction

Oral cancer is the most persistent, aggressive primary malignant sarcoma that is globally prevalent. Though chemotherapy is the only treatment option, it has not progressed for years to overcome its detrimental side effects. Introducing novel therapeutic techniques to improve effectiveness is the need of the hour.

Aim

This study aimed to investigate the pro-apoptotic effects of naringin in oral cancer cell lines.

Methodology

The cell viability of oral cancer cells treated with naringin was measured using the 3-(4,5-dimethylthiazol-2-yl)-2,5-diphenyltetrazolium bromide (MTT) method. Naringin was given to oral cancer cells (KB-1) in concentrations ranging from 20 to 200 µM/mL for 24 hours. A phase-contrast microscope is used to examine cell morphology changes. Ethidium bromide (EtBr) staining was employed to study nuclear morphological alterations in oral cancer cells. The apoptotic nuclei were viewed under a fluorescent microscope. To determine pro-apoptotic levels, quantitative real-time polymerase chain reaction (PCR) gene expression analysis was performed to evaluate the expression of transforming growth factor-beta (TGF-β), suppressor of mothers against decapentaplegic 2 (SMAD2), tumor necrosis factor alpha (TNFα), and nuclear factor kappa B (NFκB). A scratch wound healing experiment was used to evaluate naringin's anti-migratory properties.

Results

Our study found that naringin treatment significantly reduced cell viability in oral cancer cells compared to the control group (p < 0.05). In oral cancer cells, we found an inhibitory concentration (IC_50_) of 125.3 μM/mL. Following treatment, fewer cells were present, and those that were present shrunk and displayed cytoplasmic membrane blebbing. The EtBr staining reveals chromatin condensation and nuclear breakage in treated cells. The study found that naringin downregulates the expression of B-cell leukemia/lymphoma 2 (Bcl-2), TGF-β, SMAD2, TNFα, and NFκB and upregulates the expression of Bcl-2-associated agonist of cell death (BAD), Bcl-2-associated protein X (BAX), and caspase-3. Furthermore, when compared to control cells, naringin significantly reduced cell migration. Naringin treatment significantly promotes apoptosis and inhibits migration by altering the SMAD2 signaling pathway.

Conclusion

Overall, this study highlights the promising role of naringin as a pro-apoptotic and cytotoxic phytochemical regulating the gene expression of Bcl-2, TGF-β, SMAD2, TNFα, NFκB, BAD, BAX, and caspase-3, thereby treating oral cancer.

## Introduction

Cancer is one of the fastest-growing diseases in the 21st century. It is distinguished by the proliferation of cells that have managed to circumvent major endogenous regulatory mechanisms [1]. The current scenario is extremely concerning, with every fourth person in the globe being in danger of developing cancer over their lifetime. Every year, more than 11 lakh new cancer cases are registered in India, whereas the global average is more than 14 million [2]. Oral cancer is the sixth most frequent carcinoma worldwide [3]. Tobacco use and smoking are the leading causes of mouth cancer. Current oral cancer treatments are extremely expensive. Oral cancer is defined as unexplained growth in the lips, cheeks, tongue, sinuses, hard and soft palates, and, in cases of oral squamous cell carcinoma (OSCC), oropharynx [4]. Oral cancer incidence worldwide is estimated to be around 275,000 each year. In the year 2018, the estimated incidence and mortality were found to be 354,864 and 177,384, respectively [5]. In India, the overall number of new oral cancer cases is believed to be around 77,000 with 52,000 deaths reported each year [6]. Males and females have approximately identical incidence and mortality rates in Japan, Indonesia, Mexico, India, Bangladesh, and Pakistan [6].

Polymerase chain reaction (PCR), tissue reflectance, autofluorescence, laser-induced autofluorescence, in vivo confocal microscopy, and DNA microarray are all being used to diagnose oral cancer. Leukoplakia, erythroplakia, oral submucous fibrosis, and lichen planus are all premalignant conditions of oral cancer [7]. Targeted drug delivery systems offer the potential to improve drug bioavailability at the primary tumor site. Oral cancer treatment options include surgery and radiotherapy. Treatment options range depending on factors such as tumor location, age, and functional results [8]. Paclitaxel, cisplatin, doxorubicin, and docetaxel and inhibitors such as cetuximab, bevacizumab, and erlotinib have demonstrated efficacy in oral cancer patients [9]. Infection, hematoma, and skin necrosis are some of the downsides of current therapeutic approaches. The downsides of radiation treatments include taste changes, discomfort, mucosal ulceration, and increased saliva thickness [8]. Therapeutic medications have drawbacks such as toxicity, tiredness, and hair loss [9]. To lessen the problems of conventional treatments, we can use medications derived from plants, known as phytochemicals. They are nonnutritive compounds found in plants that help to treat cancer [10]. Paclitaxel, etoposide, vincristine, and naringin are all plant-based anticancer medicines. They are made with a variety of organic ingredients, including whole grains, fruits, vegetables, nuts, and herbs.

Naringin is a bioactive polyphenol found in citrus fruits that is a natural dihydro-flavanone [11]. Numerous studies have shown that the antioxidant property can protect against inflammation and cancer. Naringin can inhibit cancer growth in a variety of body parts by acting as an effective alternative treatment. Naringin (4',5,7-trihydroxyflavanone-7-rhamnoglucoside) is a flavonoid compound found in citrus fruits such as lemon, orange, mandarin, and grapefruit. Recently, plant-based chemical compounds known as phytochemicals have demonstrated promising results in cancer treatment. The anticancer activity of these chemicals may derive from their usage in monotherapy or in conjunction with chemotherapy medicines [12]. Naringin (molecular formula: C_27_H_32_O_14_) has a typical flavonoid structure, with three rings (two of which are benzene rings) joined by a three-carbon chain and two rhamnose units attached at the C7 position. Naringin's antioxidant action is due to the 7-OH, 4′-OH, and 5-OH groups, as well as the 4(=O) carbonyl group on the C ring and the 5-OH group on the A ring. Naringin has a molecular weight of 580.54 [13]. Water, alcohol, acetone, and heated acetic acid all dissolve naringin. They take on a crystalline structure. Naringin's storage temperature ranges from 2°C to 8°C. Citrus fruits include a flavone glycoside with unique pharmacological and biological properties [14]. Naringin's effects on apoptosis involve targeting both extrinsic and intrinsic apoptosis pathways. It can cause apoptosis by upregulating p53 expression, cleaving B-cell leukemia/lymphoma 2 (Bcl-2)-associated protein X (BAX) and caspase-3, suppressing B-cell leukemia/lymphoma 2 (Bcl-2), and surviving in the SGC-7901 cell line. Their anticarcinogenic effects are mediated by a variety of cell signaling mechanisms [15]. The overexpression of tumor necrosis factor (TNF) family members has been linked to the extrinsic apoptotic pathway. It also stopped glioblastoma cells from migrating or invading. They have a high therapeutic potential due to their antioxidative, anti-inflammatory, and anti-apoptotic effects. They influence human pathophysiology. Naringin enhances antioxidant defenses and hence protects against oxaliplatin (OXL)-induced testicular damage [16].

Naringin has been studied in several cancers, including glioblastoma and ovarian cancer. However, studies investigating naringin's impact on oral cancer are currently scarce. This study intends to analyze the anticancer potential of naringin on oral cancer cells in vitro.

## Materials and methods

Cell line maintenance

Human oral cancer cell lines (KB-1) were obtained from the National Centre for Cell Science (NCCS), Pune. Cells were grown in T25 culture flasks using Dulbecco's modified eagle medium (DMEM) supplemented with 10% fetal bovine serum (FBS) and 1% antibiotics (penicillin-streptomycin). To maintain the cells at 37°C, a humidified environment with 5% CO_2_ was used. Once 80% confluence was achieved, the cells were trypsinized and passaged.

Cell viability (3-(4,5-dimethylthiazol-2-yl)-2,5-diphenyltetrazolium bromide {MTT}) assay

The cell viability of the naringin-treated oral cancer cell line was determined using the 3-(4,5-dimethylthiazol-2-yl)-2,5-diphenyltetrazolium bromide (MTT) test. The assay relies on metabolically active cells converting soluble yellow tetrazolium salt to insoluble purple formazan crystals. Cell lines were grown in 96-well plates at 5×10^3^ cells per well. Twenty-four hours after plating, cells were washed twice with 100 μL serum-free media and starved for three hours at 37°C. Following starvation, cells were treated with various concentrations of naringin (20-200 μM/mL) for 24 hours. The medium from the control and naringin-treated cells was withdrawn after treatment, and 100 μL of DMEM containing MTT (0.5 mg/mL) was added to each well. For four hours, cells were maintained in a CO_2_ incubator at 37°C. After removing the MTT-containing media, the cells were washed with 1X phosphate-buffered saline (PBS). Following a one-hour incubation period in the dark, 100 μL of dimethyl sulfoxide (DMSO) was used to dissolve the formazan crystals. Next, a micro-enzyme-linked immunosorbent assay (ELISA) plate reader set to 570 nm was used to measure the intensity of the color. As a proportion of control cells grown in media without serum, the number of viable cells was expressed. In the control media, there was no treatment, and cell viability was 100%. The formula for calculating cell viability is % cell viability=[A570 nm of treated cells/A570 nm of control cells]×100.

Study of morphology

The MTT assay was used to identify the optimal dosage for the oral cancer cell line KB-1, which would be employed in further analyses. A phase-contrast microscope is used to examine changes in cell morphology. Naringin was applied for 24 hours after planting 2×10^5^ cells in six-well plates. At the end of the incubation period, the medium was removed from the cells and cleansed again with phosphate-buffered saline (PBS pH 7.4). Plates were inspected with a phase-contrast microscope.

Staining using ethidium bromide (EtBr) to identify the mode of cell death

EtBr staining was employed to evaluate naringin's effect on oral cancer cell mortality. Following a 24-hour Naringin treatment, the cells were washed with ice-cold PBS. The pellets were resuspended using 5 µL of EtBr (1 mg/mL). A fluorescent microscope was then used to observe the tagged cells' apoptotic changes.

Scratch wound healing test

Oral cancer cells (2×10^5^ per well) were placed in six-well culture plates. Under an inverted microscope, a 200 μL tip was used to make an incision in the cell monolayer, followed by a wash with PBS. After administering the 50% inhibitory concentration (IC_50_) dose and serum-free culture media to the control cells for 24 hours, the damaged area was imaged under the same microscope. In addition, the studies were repeated three times for each treatment group.

Real-time PCR

Real-time PCR was utilized to analyze gene expression of Bcl-2-associated agonist of cell death (BAD), Bcl-2-associated protein X (BAX), caspase-3, B-cell leukemia/lymphoma 2 (Bcl-2), transforming growth factor-beta (TGF-β), suppressor of mothers against decapentaplegic 2 (SMAD2), tumor necrosis factor alpha (TNFα), and nuclear factor kappa B (NFκB) signaling molecules and others. Total RNA was isolated using a standardized technique and Trizol Reagent (Sigma-Aldrich, Burlington, MA). A PrimeScript 1st Strand cDNA Synthesis Kit (TaKaRa, Kusatsu, Japan) was used to synthesize cDNA from 2 μg of RNA. To amplify the desired genes, specific primers were utilized. The PCR reaction was carried out using GoTaq® qPCR Master Mix (Promega, Madison, WI), which contains all the PCR components, as well as SYBR green. The real-time PCR was performed using Biorad CFX96 PCR (Hercules, CA) equipment. The comparative CT approach was used to analyze the findings, and the fold change was estimated using Schmittgen and Livak's (2008) 2-∆∆CT method.

Statistical analysis

In SPSS (IBM SPSS Statistics, Armonk, NY), all collected data were analyzed in triplicate using one-way ANOVA and then Student's t-test. P<0.05 was determined to be the statistically significant level.

## Results

Naringin's cytotoxic and pro-apoptotic properties on the KB-1 oral cancer cell line

**Figure 1:**
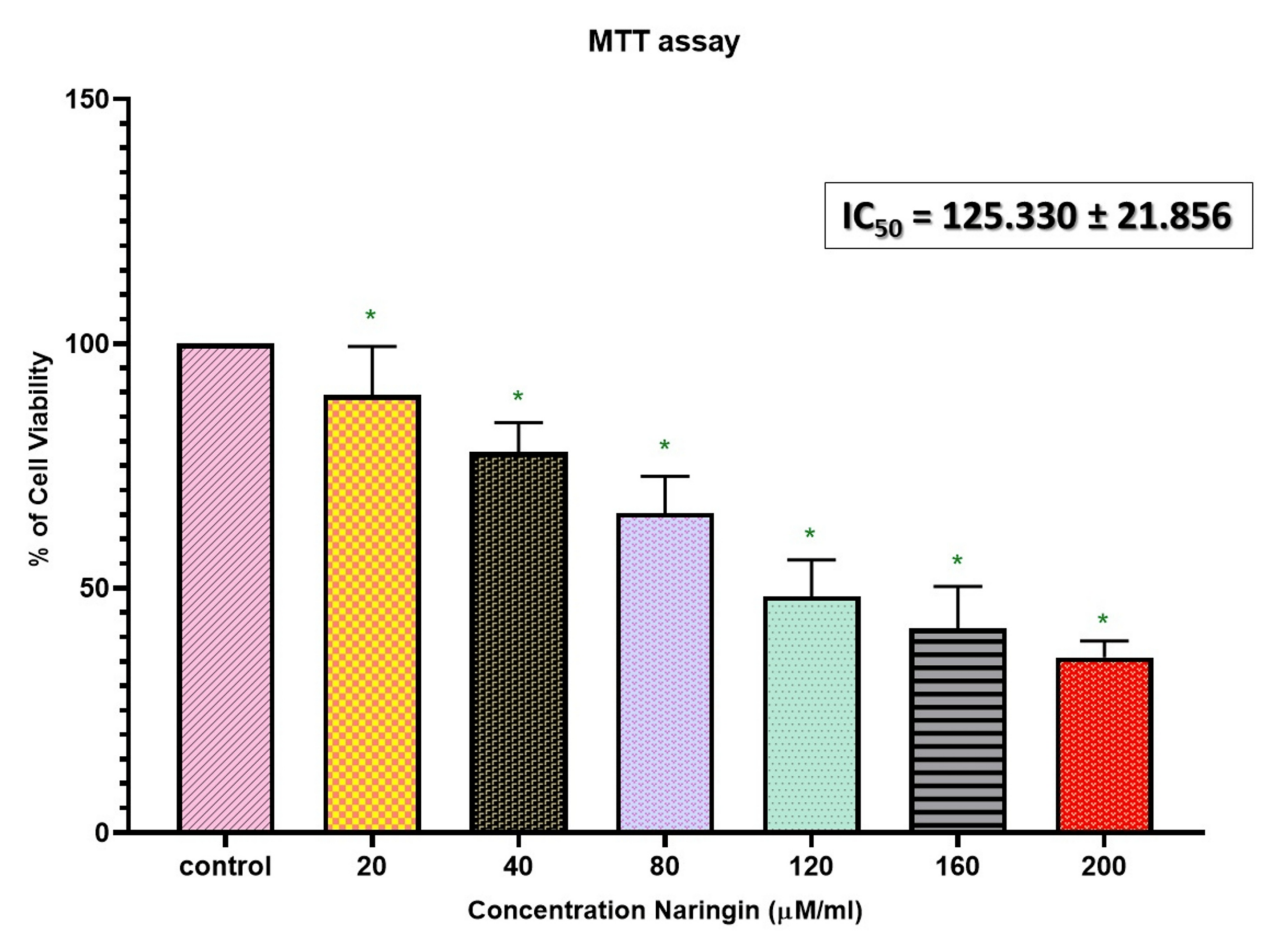
Naringin's cytotoxic effects on oral cancer cells The MTT test was used to assess the viability of the cells after they were treated with naringin (20-200 μM/mL) for 24 hours. The data are displayed as mean±SD (n=3) *A P-value of <0.05 indicates the statistical significance between the treatment group and the control group MTT, 3-(4,5-dimethylthiazol-2-yl)-2,5-diphenyltetrazolium bromide; IC_50_, 50% inhibitory concentration; SD, standard deviation

For 24 hours, the cells were subjected to a range of doses (20-200 µM/mL). Figure 1 illustrates the reduction in the sustainability of the naringin-treated KB-1 oral cancer cell line when compared to the control. Viable cell count decreased as concentration increased. At a dosage of 125.3 µM/mL, our results demonstrated a 50% growth inhibition, which was approved as the inhibition level (IC_50_) dosage value and used for further study.

Morphological alterations induced by naringin on oral cancer cells (KB-1)

**Figure 2:**
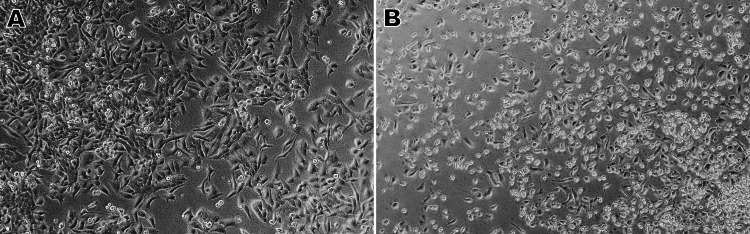
Effect of naringin (125.3 μM/mL) on the morphology of human KB1 oral cancer cells After being exposed to naringin (125.3 μM/mL) for 24 hours, the cells were examined using an inverted phase-contrast microscope. (A) Control cells without naringin treatment. (B) Cells after 24 hours of treatment with naringin (125.3 μM/mL) showing cell shrinkage and cytoplasmic membrane blebbing

Compared to the untreated cells, the treated KB-1 oral cancer cell line showed significant morphological changes after receiving 125.3 µM/mL of naringin extract for 24 hours (Figure 2A, 2B). Apoptotic cells are indicated by many alterations, including reduced cell count and the atrophy of the cells. Additional morphological changes such as the loss of contact with neighboring cells were observed in dying cells. Furthermore, a few delicate cells were taken off from the plate surface.

Identification of apoptotic cells by EtBr staining

**Figure 3:**
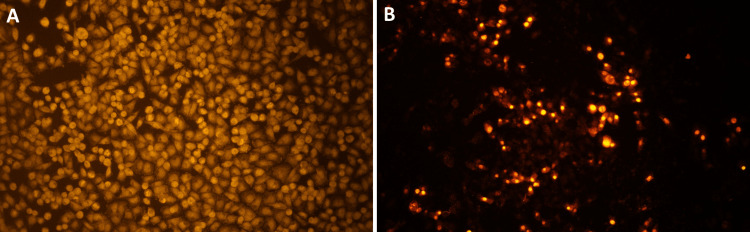
Detection of apoptotic cells in naringin (125.3 µM/mL)-treated oral cancer cells by EtBr staining Human oral cancer cells were treated with naringin for 24 hours along with the control group. After treatment, the cells underwent EtBr staining incubation. An inverted fluorescence microscope was utilized for capturing pictures. (A) Control cells without naringin treatment. (B) Cells after 24 hours of treatment with naringin (125.3 μM/mL) EtBr: ethidium bromide

Following a 24-hour exposure to 125.3 µM/mL of naringin, the nuclear morphology of apoptotic cells is examined using EtBr staining. A fluorescent microscope was used to observe the treated cells after they had been stained for 20 minutes with EtBr dye. The results showed that even though EtBr only detected membrane-weakened cells, the orange- and yellow-stained cells represent the start and finish of apoptosis, respectively (Figure 3A, 3B). The results demonstrate that naringin extract causes apoptosis in KB-1 oral cancer cell lines.

Influence of naringin on apoptotic gene expression

**Figure 4:**
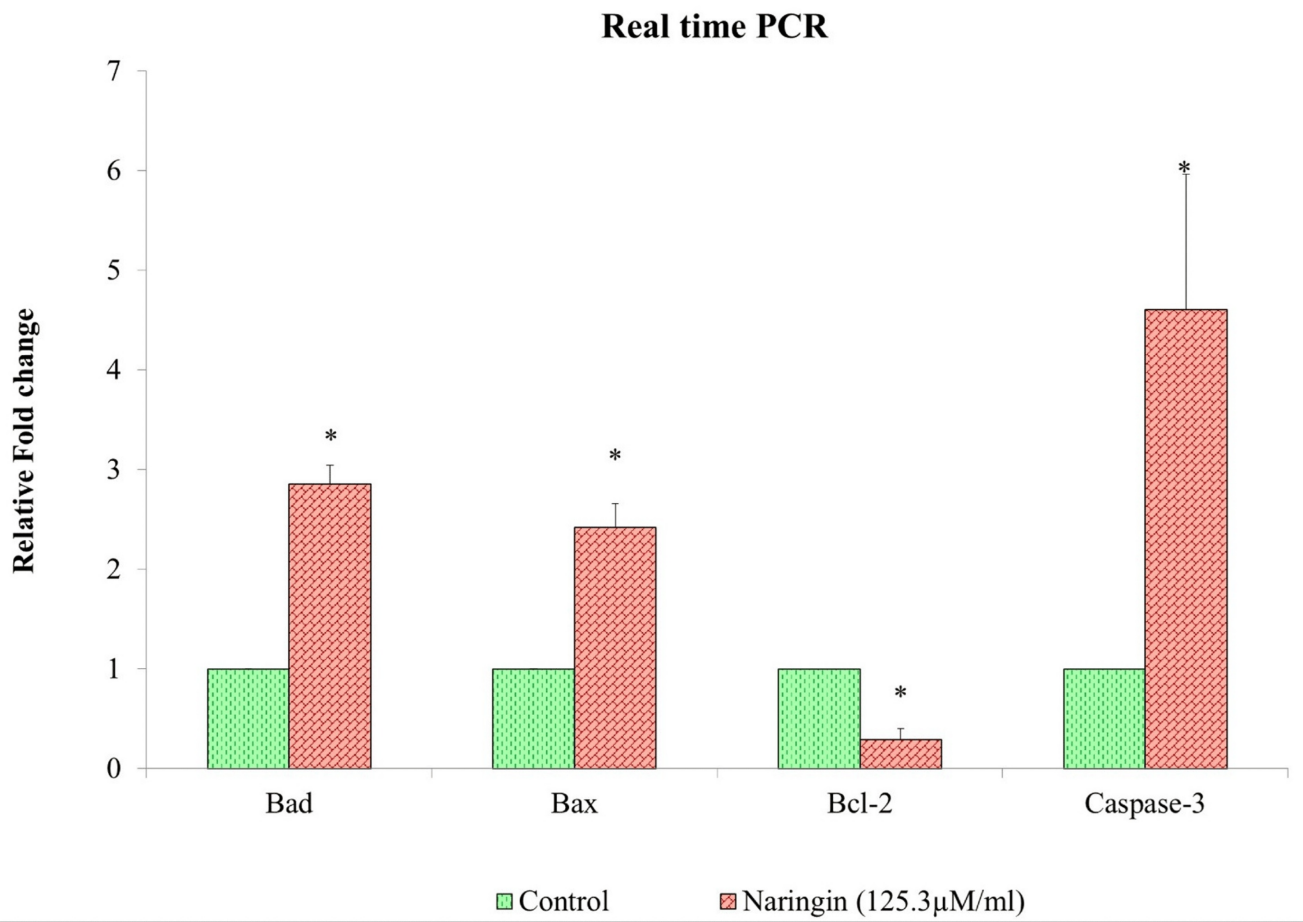
Effect of naringin on BAD, BAX, Bcl-2, and caspase-3 gene expression in oral cancer After normalizing target gene expression to GAPDH mRNA expression, the results are presented as a fold change from control. Each bar represents the mean±standard deviation of three different observations *A P-value of <0.05 indicates statistical significance between the drug-treated and control groups Bcl-2, B-cell leukemia/lymphoma 2; BAD, Bcl-2-associated agonist of cell death; BAX, Bcl-2-associated protein X; GAPDH, glyceraldehyde 3-phosphate dehydrogenase

The genes BAD, BAX, caspase-3, and Bcl-2 are thought to be markers specific to KB1 oral cancer cells. Since anti-apoptosis gives cancer cells the ability to migrate and invade, it is believed to be an essential stage in their metastasis. In oral cancer cells, we found that treatment with naringin markedly reduced Bcl-2 and increased the expression of the anti-apoptotic genes BAD, BAX, and caspase-3 (Figure 4). The inhibition of oral cancer cell proliferation and migration is associated with the regulation of apoptotic genes.

Anti-metastatic potential of naringin

**Figure 5:**
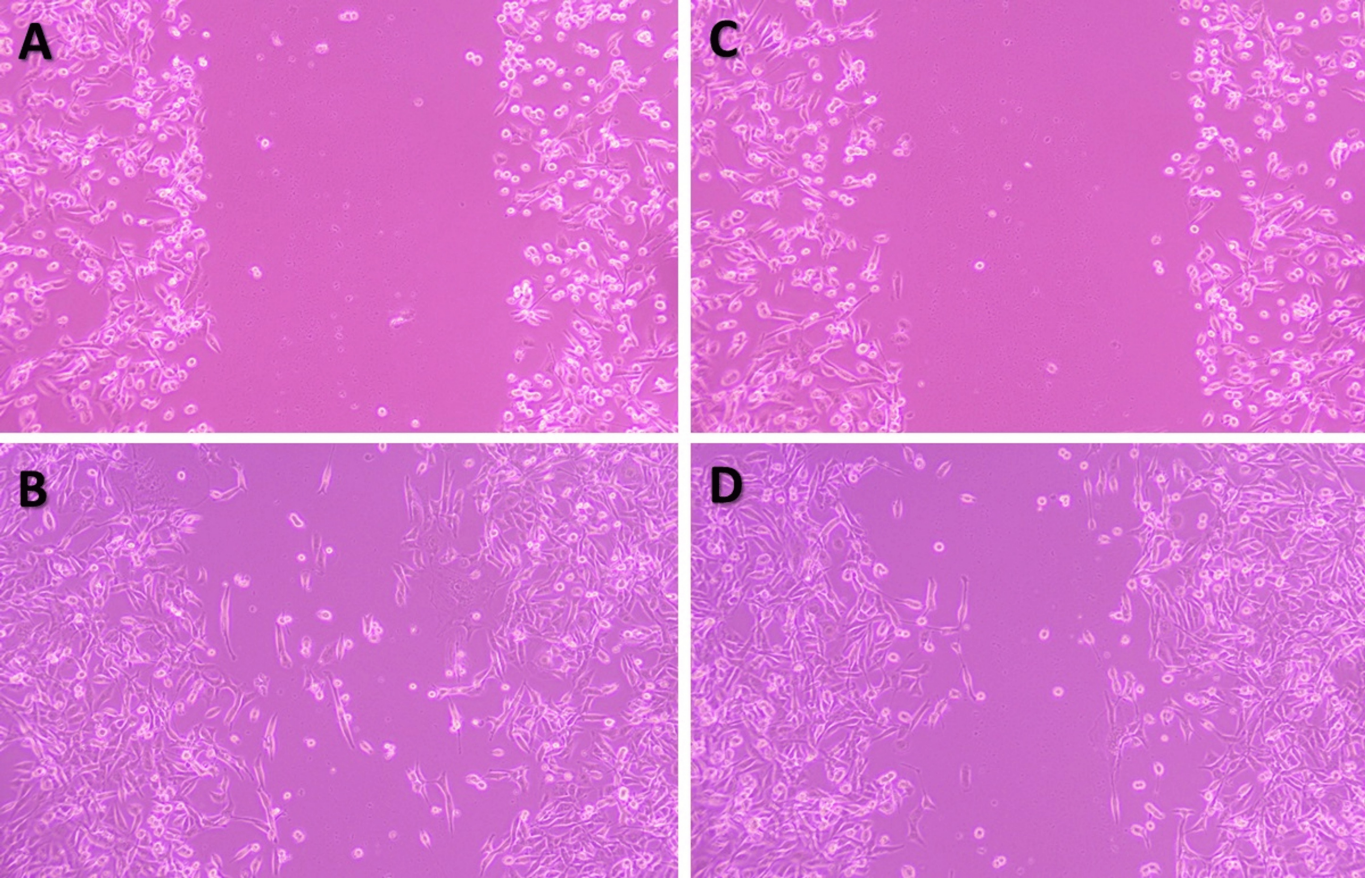
Scratch wound healing assay An in vitro scratch wound healing experiment was utilized to assess naringin's anti-migratory properties. After damaging oral cancer (KB-1) cells, a 24-hour cell migration experiment was performed with and without naringin (125.3 µM/mL) treatment. The images were obtained using an inverted phase-contrast microscope. (A) Cells without naringin treatment at zero hours after scratching. (B) Deliberately migrated cells without naringin treatment, 24 hours after scratching. (C) Naringin (125.3 μM/mL)-treated cells at zero hours after scratching. (D) Naringin (125.3 μM/mL)-treated cells at 24 hours after scratch introduction showing restricted migration

Cancer cells have the potential to migrate, which is an essential part of tumor invasion and spread. By altering their cytoskeletal dynamics and adhesion properties, cancer cells can migrate, enabling them to break apart from the main tumor and infect neighboring organs. The investigation of cell migration and metastasis within a controlled cell culture environment is crucial in identifying potential targets for treatment and creating innovative approaches to combat cancer. A scratch test was used to assess how naringin affected the migration of oral cancer cells. The results showed that naringin lowers the rate of cell migration when compared to control cells. Untreated cancer cells in the control group spread over nearly half of the area they had scratched after a day. However, naringin (125.3 μM/mL)-treated oral cancer cells showed inhibited migration. Figure 5A-5D shows a decrease in the cell migration distance between the naringin-treated group and the control group.

Naringin's influence on TNFα/NFκB and TGF-β/SMAD2 gene expression****

**Figure 6:**
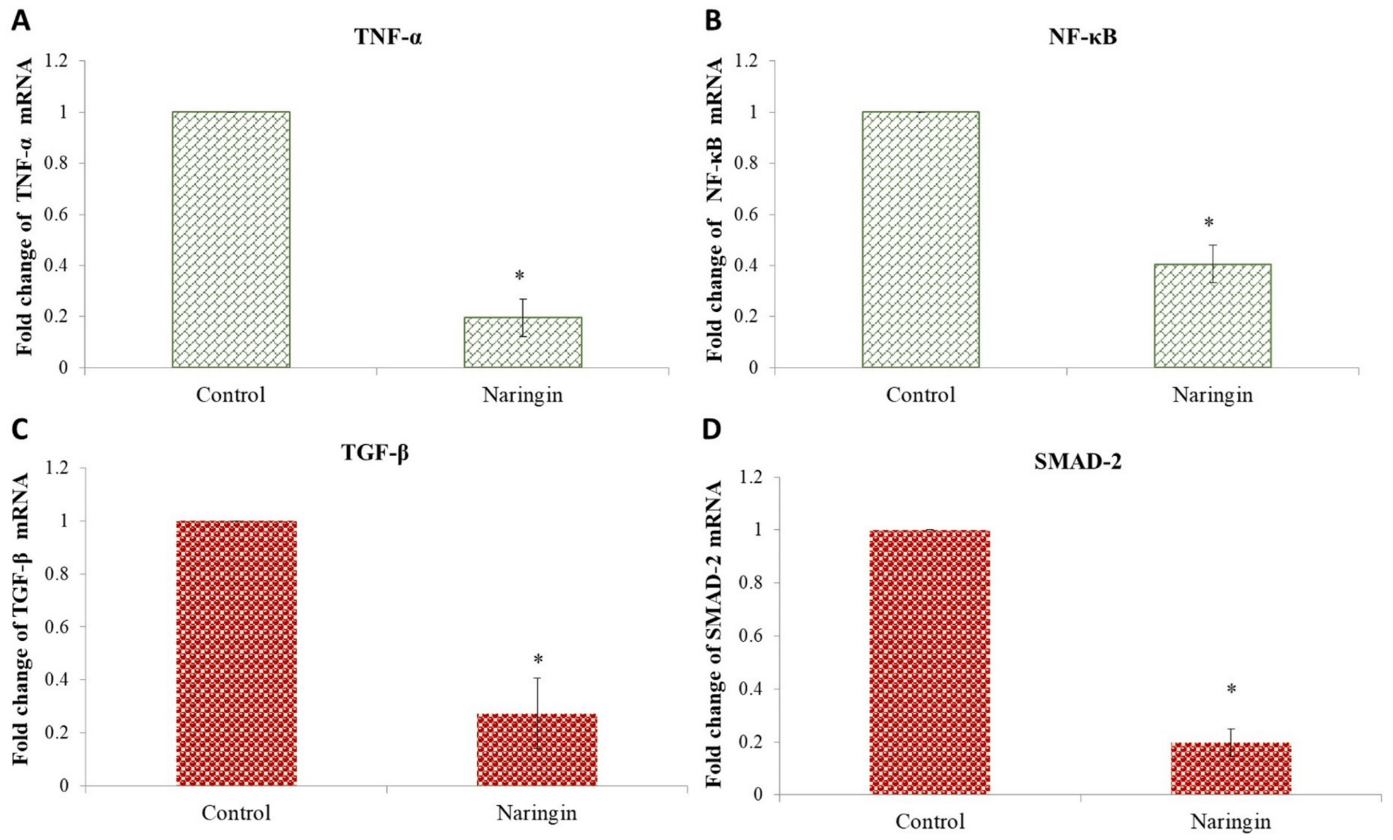
Effect of naringin (125.3 μM/mL) on the gene expression of (A) TNFα, (B) NFκB, (C) TGF-β, and (D) SMAD2 in oral cancer cell After normalizing target gene expression to GAPDH mRNA expression, the results are presented as a fold change from control. Each bar represents the mean±standard deviation of three different observations *A P-value of <0.05 indicates statistical significance between the drug-treated and control groups TNFα, tumor necrosis factor alpha; NFκB, nuclear factor kappa B; TGF-β, transforming growth factor-beta; SMAD2, suppressor of mothers against decapentaplegic 2; GAPDH, glyceraldehyde 3-phosphate dehydrogenase

We then examined the mRNA expression of transforming growth factor-beta (TGF-β), suppressor of mothers against decapentaplegic 2 (SMAD2), tumor necrosis factor alpha (TNFα), and nuclear factor kappa B (NFκB) signaling molecules in oral cancer cell lines. Oral cancer cell development and metastasis are strongly impacted by the TGF-β, SMAD2, TNFα, and NFκB signaling pathways. Oral cancer cells contain TGF-β, a vital regulator of angiogenesis that stimulates the formation of malignancies. The progression of oral cancer cells is also associated with SMAD2 signaling. The anomalous activation of TGF-β, SMAD2, TNFα, and NFκB signaling in oral cancer cells can encourage the growth and spread of tumors. Treatment strategies that target downstream signaling components or TGF-β, SMAD2, TNFα, and NFκB receptors are being researched to limit the spread of cancer. We employed real-time PCR to assess TGF-β/SMAD2 mRNA expression in KB-1 cell lines. We discovered that naringin treatment dramatically reduced TGF-β, SMAD2, TNFα, and NFκB gene expression in oral cancer cells. TGF-β, SMAD2, TNFα, and NFκB downregulation was associated with a decrease in oral cancer cell movement (Figure 6A-6D).

## Discussion

A copious number of studies have been conducted in the last several years in search of novel chemotherapeutics. Herbal plants hold great potential as a source of therapeutic targets for a range of acute and chronic conditions. The surgical removal of solid cancers, though effective, possesses a high chance of recurrence [17]. Only a small number of patients can benefit from current cancer therapy. This is because each tumor is unique and not all tumors are brought on by the same genetic abnormalities [18]. The creation of new compounds for therapeutic application has not significantly risen despite efforts in drug discovery. Despite being a fairly rare disease, oral cancer cells have a five-year survival rate and poor treatment outcomes.

This work focuses on two of the many impacts naringin has on oral cancer cell lines: pro-apoptotic and antiproliferative effects through the control of signaling pathways. By investigating the intricate molecular processes underlying these outcomes, the research illuminates potential medicinal applications of naringin in the management of oral cancer cells. The results obtained yield an important novel knowledge of the apoptotic pathways that naringin regulates, which further advances our understanding of its anticancer actions [16].

In this study, we examined naringin's cytotoxic and pro-apoptotic effects on the oral cancer cell line KB-1. Initially, the oral cancer cell line was exposed to a range of naringin concentrations (20-200 μM/mL) for 24 hours to evaluate the drug's growth-inhibiting capabilities. Our findings demonstrated that naringin administration significantly decreased KB-1 cell viability in a dose- and time-dependent manner. Naringin significantly induced cell death at higher concentrations (125.3 µM/mL), suggesting a potential cytotoxic action. The anticancer potential of the morphology was verified using phase-contrast microscopy, and an IC_50_ value of 125.3 μM/mL was selected to evaluate the inhibitory effect further. When naringin was administered, the number of oral cancer cells significantly decreased. These cells also showed cytotoxic effects such as cytoplasmic membrane blebbing and cell shrinkage.

Apoptosis, also known as programmed cell death, is represented by DNA breakage, chromatin condensation, cell shrinkage, and the activation of certain enzymes known as caspases [19]. The apoptotic process has been ceased by cancer to spread. Inducing apoptosis in tumor cells is the most well-established anticancer technique used in numerous cancer treatments [10,20].

Our results show that naringin therapy has a pro-apoptotic effect because it significantly increases the proportion of apoptotic cells. EtBr staining was used to confirm the presence of naringin-induced apoptotic cells in oral cancer cell lines. In recent times, many studies have been based on naringin's anticancer potential over various cancer cells. While naringin itself can still be used as a lead chemical for the development of potential anticancer medicines, its analogs can be produced to build new pharmaceuticals with improved pharmacological properties [21]. One of the main drawbacks of studying Naringin's anticancer effect in vitro is its incapacity to faithfully replicate the complex connections between many organs and the immune system found in the human body. Consequently, promising in vitro results may not be a reliable predictor of naringin's safety and efficacy in living organisms. In vivo investigations and additional investigation into the molecular mechanism underlying naringin's anticancer action are necessary to attain a more comprehensive understanding of the drug's therapeutic potential. These investigations will contribute to bridging the knowledge gap that exists between laboratory findings and real-world use in cancer therapy.

Limitations

In vitro anticancer activity studies of naringin have limitations; these studies often fail to replicate the complexities of the human body's environment, including interactions with the immune system and various organs. Consequently, promising results observed in vitro may not accurately predict the efficacy and safety of naringin when used in living organisms. For a more comprehensive understanding of the therapeutic potential of naringin, further research involving understanding the molecular mechanism of anticancer activity and in vivo studies is essential to bridge the gap between laboratory findings and real-world application in cancer treatment.

## Conclusions

This study investigated the impact of naringin on oral cancer cell line mediated by the TGF-β, SMAD2, TNFα, and NFκB signaling pathways. The study's in vitro analysis revealed the anticancer and anti-migratory potential of naringin and its hidden role in influencing significant apoptotic signaling pathways by regulating the gene expression of TGF-β, SMAD2, TNFα, and NFκB genes. These findings provide knowledge on the clinical applications of naringin in treating oral cancer cells, adding to the growing body of information about natural compounds as potential adjuvants in cancer therapy. In summary, this research emphasizes the potential of naringin as a cytotoxic and pro-apoptotic phytochemical that modulates the expression of Bcl-2, TGF-β, SMAD2, TNFα, NFκB, BAD, BAX, and caspase-3 genes, ultimately aiding the treatment of oral cancer. In recent years, various phytochemicals have been widely contributing to cancer therapeutics. With further preclinical and clinical tests, naringin could play a significant role in oral cancer treatment.
